# A Comparative Study of Antifungal Activity of Endodontic Irrigants

**Published:** 2015-03-18

**Authors:** Zahed Mohammadi, Saeed Asgary

**Affiliations:** a *Iranian National Elites Foundation, Tehran, Iran;*; b * Iranian Center for Endodontic Research, Research Institute of Dental sciences, Dental School, Shahid Beheshti University of Medical Sciences, Tehran, Iran*

**Keywords:** *Candida Albicans*, Chlorhexidine, Chlor-Xtra, Hypoclean, MTAD, NaOCl, Sodium Hypochlorite, Tetraclean

## Abstract

**Introduction: **The purpose of this *in vitro* study was to assess the antifungal activity of final canal rinse with either three concentrations of sodium hypochlorite (NaOCl) (0.5, 2.6 and 6%), two concentrations of chlorhexidine (CHX) (2% and 0.2%), MTAD, Tetraclean, Hypoclean and Chlor-Xtra on *Candida albicans* (C. *albicans*) in a human tooth model. **Methods and Materials: **Two hundred and thirty five extracted human maxillary central and lateral incisors were used in this study. Teeth were randomly divided into nine test groups (*n*=25) and positive and a negative control groups (*n*=5). After cleaning and shaping, teeth were contaminated with C. *albicans *and incubated for 72 h. The irrigation solution in nine experimental groups included: 6% NaOCl, 2.6% NaOCl, 0.5% NaOCl, 2% CHX, 0.2% CHX, MTAD, Tetraclean, Hypoclean and Chlor-Xtra. After culturing on Sabouraud 4% dextrose agar, colony-forming units (CFU) were counted. **Results: **6% NaOCl, 2% CHX and Chlor-Xtra were equally effective (*P*>0.05) and significantly superior to MTAD and Tetraclean (*P*<0.05). In addition, the effectiveness of Tetraclean and MTAD was significantly less than Hypoclean, NaOCl at all concentrations (6% 2.6% and 0.5%), MTAD and 0.2% CHX (*P*<0.05). Furthermore, Tetraclean was significantly more effective than MTAD (*P*<0.05). **Conclusion**: Antifungal activity of 6% NaOCl, Chlor-Xtra and 2% CHX was significantly greater than 2.6% NaOCl, 0.5% NaOCl, MTAD, 0.2% CHX and Tetraclean.

## Introduction

Microorganisms have an essential role in induction and continuing of pulp and periapical pathosis [1-3]. In an elegant study on rats, Kakehashi *et al.* [1] revealed the healing of the pulps exposed to the oral environment in germ-free animals, whereas bacterially-contaminated pulps revealed complete necrosis. Furthermore, Sundqvist [2] showed that all cases with periapical radiolucency had microbes in the root canal system and there were always 2 to 12 bacterial strains present. 

Among root canal microbiota, fungi play an important role in failure of root canal treatment [4]. Fungi are common opportunistic pathogens in the oral cavity. It seems that one-third of healthy individuals carry fungi in their normal flora [5]. The genus *Candida albicans *(*C. albicans*) is the most important and influential one [5]. The incidence of *C. albicans* in the oral cavity of the healthy adults and patients infected with human immunodeficiency virus are reported to be 30-45% [6, 7] and 95% [8], respectively. Different identification methods (such as culturing, molecular genetics and *in situ* electron microscopy) have revealed that fungi are more common in secondary endodontic infections than primary ones [4, 5]. Even calcium hydroxide, as the most common intracanal medicament, is not effective in killing *Candida* species [9].

Sodium hypochlorite (NaOCl) is the most common root canal irrigant with both antimicrobial and tissue-dissolving properties [10]. It has been demonstrated that 0.5% NaOCl kills *C. albicans* during a 10-sec contact time [10]. However, one of the major drawbacks of NaOCl in the root canal system is its high surface tension, which limits its penetration into dentinal tubules and irregularities of the root canal system [11]. 

In order to overcome the high surface tension of NaOCl, two NaOCl-based root canal irrigants have been introduced. Chlor-Xtra is a mixture of 6% NaOCl with powerful wetting agents and proprietary surface modifiers. Alkylating agents have also been added to increase the electrical capacity of the solution[12]. It is stated that Chlor-Xtra has 2.5 times more wettability and significantly more tissue dissolving activity than standard NaOCl. Furthermore it is significantly more stable and does not decompose as fast as NaOCl because normal NaOCl stays on the surface of the protein molecule and rapidly converts to HCl, whereas Chlor-Xtra penetrates and does not convert to HCl [12].

Hypoclean (Ogna Laboratori Farmaceutici, Muggiò, Italy) is another NaOCl-based solution which is composed of 5.25% NaOCl and two detergents [13]. Studies on Hypoclean are very limited. Its 28-day substantivity has been demonstrated [13]. Another study showed that the surface tension of Hypoclean was significantly lower than NaOCl and Chlor-Xtra [11].

Chlorhexidine (CHX) is a cationic bisguanide with a wide antimicrobial spectrum which is effective against both Gram-positive and Gram-negative bacteria as well as fungi [14]. 

There are some antibiotic-based root canal irrigation solutions in endodontics, as well. Biopure MTAD (Dentsply, Tulsa Dental, Tulsa, OK, USA) is a mixture of a tetracycline (doxycycline), an acid (citric acid), and a detergent (Tween 80) (MTAD stands for mixture of a tetracycline isomer, an acid and a detergent) [15]. It can effectively remove smear layer [15]. Furthermore, its antibacterial efficacy has been showed in several studies [16-18]. However, there are only two studies on its antifungal activity [19, 20]. Tetraclean, (Ogna Laboratori Farmaceutici, Muggiò, Italy) is also another antibiotic-based irrigant. Like MTAD, it is a mixture of an antibiotic, an acid and two detergents (propylene glycol and cetrimide). However, the concentration of the antibiotic, doxycycline (50 mg/mL) and the type of detergent (polypropylene glycol and cetrimide) differ from those of MTAD [21]. 

There is only one study on the antifungal effect of Tetraclean [20]. Furthermore, there is no study on the antifungal activity of Hypoclean and Chlor-Xtra. Therefore, it was decided to compare the *in vitro* antifungal activity of different concentrations of NaOCl (0.5%, 2.6% and 6%), CHX (0.2% and 2%), MTAD, Hypoclean, Chlor-Xtra and Tetraclean. 

## Materials and Methods

The method of this study was based on the procedure previously described by Mohammadi *et al.* [20]. Two hundred and thirty five extracted human maxillary central and lateral incisors were used. Teeth were stored in 0.2% sodium azide. A periapical radiograph was taken from each tooth to confirm the presence of a single canal and all the teeth were sectioned 14 mm from the apex. Root canal instrumentation was performed by a crown-down technique using Gates Glidden drills sizes 3 to 1 and the Mtwo rotary instrument system (VDW GmbH, Munich, Germany) to an apical size of 40 and 1 mL of 5.25% NaOCl (Merck, Darmstadt, Germany) was used between files.

After completion of instrumentation, the smear layer was removed with 1 mL of 17% EDTA (Merck, Darmstadt, Germany), followed by 3 mL of 5.25% NaOCl (Merck, Darmstadt, Germany). In order to remove the residual irrigants, the canals were flushed with 30 mL of sterile saline. Apical foramina were sealed with Cavit (Golchai, Tehran, Iran) and the teeth were coated with two layers of nail varnish. The roots were then sterilized with ethylene oxide (Kabir laboratory, Hamedan, Iran).

A suspension of *C. albicans* (ATCC 10261) was adjusted to 0.5 turbidity on the McFarland scale (1.5×10^8^ fungi/mL). Teeth were randomly divided into nine groups (*n*=25) plus a positive (*n*=5) and a negative control group (*n*=5). All teeth were inoculated with *C. albicans* except for the negative controls. The experimental teeth were placed in plastic test tube vials and 0.3 mL of the adjusted *C. albicans* suspension was injected into the canal and into the tubes to submerge the teeth. The canals of negative controls were immersed in sterile saline. The samples were incubated at 36^°^C and 91% humidity for 72 h. Every 24 h the vials containing the experimental teeth were replenished with freshly made suspensions of *C. albicans*. At 48 h, 1 μL aliquots were taken from each tooth using a calibrated inoculation loop and plated on Sabouraud 4% dextrose agar plates (Remel, Lenexa, KS, USA) to verify the growth of *C. albicans* in each sample tube. After verifying the growth of *C. albicans* in the root canals after 72 h, the final rinse was performed in each group for 5 min, as follows: 6% NaOCl (Merck, Darmstadt, Germany), 2.6% NaOCl, 0.5% NaOCl, 2% CHX (Chlorhexidina, Setubal, Portugal), 0.2% CHX, Tetraclean (Ogna Laboratori Farmaceutici, Muggiò, Italy), Hypoclean (Ogna Laboratori Farmaceutici, Muggiò, Italy), Chlor-Xtra (Vista Dental Products, Racine, WI, USA) and MTAD (Dentsply, Tulsa Dental, Tulsa, OK, USA). In positive control group infected teeth were irrigated with sterile saline and in negative control group sterile teeth were irrigated with sterile saline.

The volume of irrigants were approximately same for all samples. All specimens were irrigated with sterile 3-mL plastic syringes and 27-gauge needles until the dentin tubes were totally filled. The penetration depth of needle was up to 2 mm short of working length. Furthermore, regarding the volume of the irrigants used as final rinse, canals were completely filled with irrigants. In order to prevent the dilution of the irrigants before the experiment, excess fluid was removed from the canal with sterile paper points. In order to prevent potential carry-over effect of the irrigants, all experimental teeth were then flushed with 30-mL sterile saline. Canals were dried with sterile paper points. A small amount of sterile saline solution was introduced into the canal and agitated with a hand file approximately 1 mm short of the tooth apex.

A 1-μL inoculation loop was used to remove aliquots from the fluid in the root canal system. The aliquots were cultured on dextrose agar and the plates were incubated at 36^°^C and 91% humidity for 24 h. The number of colony forming units (CFU) of *C. albicans* served as a measure of antifungal activity. All procedures were conducted under aseptic conditions. Data were analyzed using the one-way ANOVA test.

## Results

The CFU of *C. albicans* after rinsing with the irrigant solutions are presented in the Figure 1. Findings demonstrated that 6% NaOCl, 2% CHX and Chlor-Xtra were equally effective (*P*>0.05) and were significantly superior to MTAD and Tetraclean (*P*<0.05). In addition, the effectiveness of Tetraclean and MTAD was significantly less than Hypoclean, all three concentrations of NaOCl, MTAD and 0.2% chlorhexidine (*P*<0.05). Furthermore, Tetraclean was significantly more effective than MTAD (*P*<0.05). 

## Discussion

The purpose of this study was to compare the antifungal activity of some current and three new endodontic irrigants including NaOCl (6, 2.6 and 0.5%), CHX (2 and 0.2%), MTAD, Hypoclean, Tetraclean and Chlor-Xtra. The efficacy of NaOCl and CHX against *C. albicans* is well documented [19, 22, 23]. 

A study using a dentine tube model demonstrated that presence of smear layer lead to more resistance of *C. albicans *against the antifungal effect of 0.12% CHX, 0.5% NaOCl and 5% NaOCl [22]. Another study showed that NaOCl, hydrogen peroxide, and CHX dilutions were effective against *C albicans* [23], which is in accordance to the findings of the present study. Waltimo *et al.* [24] showed that both 5 and 0.5% concentrations of NaOCl caused complete eradication of *C. albicans* cells during 30 sec, which is in contrast to the findings of the present study. In another study, Sena *et al.* [25] evaluated the effect of liquid and gel forms of 2% CHX against biofilm of* C. albicans* on cellulose nitrate membranes. The contact time required to achieve negative cultures ranged between 30 sec to 30 min. According to the findings of Ruff *et al.* [19] effectiveness of 2% CHX and 6% NaOCl against *C. albicans* was equal and was superior to 17% EDTA and MTAD. 

In another *in vitro* study, the antifungal activity of 1.3% NaOCl, 2% CHX, MTAD and Tetraclean as a final rinse against *C. albicans *was compared in a human tooth model. Findings showed that 1.3% NaOCl and 2% CHX were equally effective and significantly superior to MTAD and Tetraclean. In addition, antifungal efficacy of Tetraclean was significantly superior to MTAD [20]. MTAD and Tetraclean are two doxycycline-based root canal irrigants [26]. Considering the fact that members of Tetracycline family are bacteriostatic, the weaker efficacy of MTAD and Tetraclean compared to other irrigants can be explained. 

Considering the fact that the present study was performed on extracted teeth, the better efficacy of Tetraclean than MTAD can be explained by lower surface tension of Tetraclean (29.1 mJ/m^2^) than MTAD (31.1 mJ/m^2^) [27]. There is a reverse relation between the surface tension of root canal irrigants and their penetration into canal irregularities and dentinal tubules. 

There are a number of studies on the antibacterial efficacy of Tetraclean. Neglia *et al.* [28] indicated that Tetraclean was at least as effective as NaOCl against* Enterococcus* *faecalis *(*E. faecalis*). Furthermore, in an* in vitro *tooth model, it is showed that only in teeth irrigated with Tetraclean, the bacterial burden gradually dropped until no bacteria were detectable a few days after irrigation. Vice versa, in teeth irrigated with NaOCl, the drop in the bacterial burden was rapid but temporary and most of the teeth were recolonized after 48 h [20]. A study indicated that Tetraclean was more effective than MTAD against *E. faecalis* in planktonic culture and in biofilms of mixed species [27]. Although MTAD and Tetraclean have some antifungal activity, their weaker effect compared to NaOCl and CHX is not surprising. Based on the composition of MTAD and Tetraclean and our current understanding of *Candida*’s pathophysiology, the poorer activity of these agents could be explained. Asna Ashari *et al.* [29] showed that NaOCl was more effective against *C. albicans* compared to MTAD, which is in accordance with the findings of the present study. On the other hand Mattigatti *et al.* [30] revealed that MTAD was more effective against *C. albicans* compared to 2% NaOCl. Tirali *et al. *[31] showed that addition of calcium hydroxide to MTAD increased its antifungal activity. There is only one study on the Chlor-Xtra. Williamson *et al.* [32] evaluated the efficacy of 6% NaOCl, Chlor-Xtra, 2% CHX and CHX-Plus against biofilms of* E. faecalis *and found that 6% NaOCl and Chlor-Xtra were significantly superior. There is only one study on the antibacterial activity of Hypoclean, as well. Mohammadi *et al.* [13] demonstrated that residual antibacterial activity of Hypoclean was significantly less than Tetraclean. 

Another issue is the volume of the used root canal irrigant which was approximately similar for all samples. The treatment time chosen for final rinsing in this study (5 min) is based on another study [20]. 

## Conclusion

The effect of 6% NaOCl, Chlor-Xtra and 2% CHX on C. albicans was significantly greater than 2.6% NaOCl, 0.5% NaOCl, MTAD, 0.2% CHX and Tetraclean.
